# Predictive analytics in smart healthcare for child mortality prediction using a machine learning approach

**DOI:** 10.1515/biol-2022-0609

**Published:** 2023-07-11

**Authors:** Farrukh Iqbal, Muhammad Islam Satti, Azeem Irshad, Mohd Asif Shah

**Affiliations:** Department of Computer Science, Shaheed Zulfikar Ali Bhutto Institute of Science and Technology (SZABIST), Karachi, Pakistan; Department of Computer Science, Millennium Institute of Technology & Entrepreneurship (MiTE), Karachi, Pakistan; Faculty of Computer Science, Asghar Mall College Rawalpindi, HED, Govt. of Punjab, Pakistan; Department of Economics, Kabridahar University, Po Box 250, Somali, Ethiopia; Department of Computing and IT (DOCIT), The Millennium Universal College (TMUC), Islamabad 44000, Pakistan

**Keywords:** child mortality, developing countries, health care, predictive analytics, machine learning

## Abstract

In developing countries, child health and restraining under-five child mortality are one of the fundamental concerns. UNICEF adopted sustainable development goal 3 (SDG3) to reduce the under-five child mortality rate globally to 25 deaths per 1,000 live births. The under-five mortality rate is 69 deaths per 1,000 live child-births in Pakistan as reported by the Demographic and Health Survey (2018). Predictive analytics has the power to transform the healthcare industry, personalizing care for every individual. Pakistan Demographic Health Survey (2017–2018), the publicly available dataset, is used in this study and multiple imputation methods are adopted for the treatment of missing values. The information gain, a feature selection method, ranked the information-rich features and examine their impact on child mortality prediction. The synthetic minority over-sampling method (SMOTE) balanced the training dataset, and four supervised machine learning classifiers have been used, namely the decision tree classifier, random forest classifier, naive Bayes classifier, and extreme gradient boosting classifier. For comparative analysis, accuracy, precision, recall, and *F*1-score have been used. Eventually, a predictive analytics framework is built that predicts whether the child is alive or dead. The number under-five children in a household, preceding birth interval, family members, mother age, age of mother at first birth, antenatal care visits, breastfeeding, child size at birth, and place of delivery were found to be critical risk factors for child mortality. The random forest classifier performed efficiently and predicted under-five child mortality with accuracy (93.8%), precision (0.964), recall (0.971), and *F*1-score (0.967). The findings could greatly assist child health intervention programs in decision-making.

## 1 Introduction

The World Health Organization (WHO) and United Nations International Children’s Emergency Fund (UNICEF), in 2004, established an Inter-Agency Group for the approximation of child mortality (UNIGME) and for sharing data on mortality of children, estimation of mortality rates of children with improved techniques, provide insights into the improvement in achieving child survival targets and improve country capacity for producing timely and reliable under-five mortality estimates. Child deaths can be handled with naive approaches such as improving mothers’ education, providing clean water, and appropriate treatment by health care providers. A considerable reduction can be noted globally in deaths of under-five children; however, it is a fundamental health problem in underdeveloped countries. The latest international figures indicate that there were 5.3 million under-five deaths in 2018, which can be considered as 15,000 deaths per day [1,2,3].

Universally, the death rate for males and females used to be “41” and “36” deaths for every 1,000 live births. About 2.9 million males and 2.4 million females lost their lives before reaching the age of five, uncovered in the UNICEF Report 2019. Lowering the death of children in developing countries is one of the main obstacles. For the accomplishment of sustainable development goals (SDGs), more than 50 countries need to accelerate reductions in under-five mortality of children. Democratic Republic of Congo, Pakistan, Nigeria, and India contributed to half of all under-five child deaths in 2018. Following the SDGs, the aim is the reduction of the under-five death rate of children by 2030 to at least 25 per 1,000 live births.

In the last six decades, since its independence, Pakistan has made substantial economic growth, as measured by some main social indicators. Facilities for health and education have been extended and strengthened, and life expectancy has increased. Child mortality rates have dropped, yet the world’s seventh most crowded nation on the planet, with approximately 220 million inhabitants, faces serious problems in the maternal and child health sector [4,5,6,7,8]. Child death rates are a major problem in developing countries, especially in Pakistan, which needs more attention. The death rate of under-five children is still high in Pakistan despite various package-based programs being introduced in Pakistan. According to Pakistan Demographic Health Survey, there is a decrease in the under-five child death rate, which has diminished to 74/1,000 in 2017–2018, whereas it was 112/1,000 in 1990–1991.

The Pakistan Demographic and Health Survey [9,10] captures and preserves a wide range of information. To facilitate health professionals in recovering helpful knowledge and information from these huge datasets, a novel generation of computational approaches and tools is urgently needed. Predictive analytics is a domain that examines historical and real-time data to generate predictions using diverse techniques such as modeling, data mining, statistics, and machine learning. Predictive analytics gives clinicians, financial analysts, and administrative staff a heads-up on potential conditions before they occur, allowing them to make proactive decisions about how to proceed. In the past, data mining techniques have been widely employed for medical diagnosis, financial forecasting, and credit-card fraud detection. Lately, these techniques have been employed for various objectives such as the identification of child mortality dynamics, locating correlations among the key parameters, and analyzing data from large datasets to identify previously unknown patterns, which it then uses to develop prediction models.

Earlier literature studies have revealed major risk factors of child mortality, which aids in determining the priority areas for intervention initiatives in order to minimize child mortality and improve child health. Moreover, a generic prediction framework is lacking for reliable assessment of child mortality employing machine learning algorithms, as well as the potential to score prominent variables and establish healthcare systems that accommodate well in developing countries.

Therefore, the goal of this study is to create a predictive framework that health professionals may use to forecast child mortality in order to make timely interventions and possibly reduce elements that cause high mortality rates. The validation of our contributed framework is conducted using the Pakistan demographic health survey dataset. Supervised machine learning classifiers, like random forest (RF), decision tree (DT), naive Bayes (NB), and extreme gradient boosting (XGB) have been verified on real-world datasets.

### 1.1 Contribution

The main contribution of this study is illustrated below:

- This proposed work contributes to improving childhood health by analyzing the risk factors in child mortality with the help of an automated feature selection method, the information gain, and a predictive analytics framework for the prediction of child mortality.
- With the help of machine learning algorithms such as DT, RF, NB, and XGB after being tested on Pakistan demographic and health survey dataset, we evaluate the efficacy of RF with 93.8% accuracy which surpassed the other classifiers.

### 1.2 Scheme organization

In this article, Section 2 describes the literature review of the related studies. Section 3 presents the methods used in this study for the development of the framework. Section 4 demonstrates the results and comparative analysis. Section 5 concludes this article.

## 2 Literature review

We examined thoroughly interrelated work and highlight the benefits and curbs of previously described methodologies. Furthermore, we only evaluated articles that analyzed data from developing countries and used automated methods for the estimation of child mortality.

In Tesfaye et al. [9], the data mining approach employed to develop a web-based child mortality estimation model is in the Ethiopian language. The Ethiopian demographic and health survey dataset was used for training and testing DT and PART algorithms. Statistical Package for the Social Sciences (SPSS) was used for the analysis and Waikato environment for knowledge analysis (WEKA) for the implementation of data mining algorithms. Performance evaluation metrics such as accuracy, precision, and recall were used to evaluate the performance of the classification models. In Ethiopia, the developed prediction model supported child health programs. Although DT and PART algorithms were employed, no decision rules were defined and addressed. Over-fitting is the major problem in DT, which can be resolved with a collection of DTs. Ensemble-based methods and feature selection methods were not tested.

Rabbani and Qayyum [11] state that Pakistan is among the countries with the highest death rate among children under the age of five and the authors looked into the major factors that influence under-five child mortality. Pakistan demographic and health survey data collected through the National Institute of Population Studies (NIPS) were used in this study and revealed that infant mortality causes high child mortality. Logistic regression and maximum likelihood estimation were used for the estimation of mortality and to develop effective strategies to overcome child deaths. Moreover, health professionals must have an understanding of local-level areas and their populations. The economic status of mothers, exposure to media, and education of mothers were found to be significant determinants to reduce child mortality.

Ahmed et al. [12] revealed the fact that child mortality in Pakistan is linked to social, economic, and environmental factors. They employed binary logistic regression to assess child mortality. Mother’s education, the interval among births, the number of members in the family, the size of the child at birth, breastfeeding, birth order, and region are all critical risk factors of child mortality in Pakistan, according to this study. Furthermore, breastfeeding on time reduces the risk of mortality in children, and when compared to other areas of Pakistan, child mortality in Baluchistan was extremely high. However, the main contribution of this research work is that it highlighted the priority areas for healthcare professionals. Unfortunately, the study’s fundamental flaw is that no advanced predictive analytics was employed to determine child mortality.

Kale [13] used a data mining-based approach to discover the reasons for children being hospitalized in the pediatric ward more recently. A case study was conducted in Nigist Eleni Mohammed Memorial Zonal Hospital with the help of famous data mining methods such as DT and artificial neural networks to discover the reasons behind children's admission to the pediatric ward. A data mining algorithm has been employed and DT produced a higher accuracy after training the model on records of the dataset. In addition, most of the children admitted to the hospital were due to lack of breastfeeding and not taking food properly according to DT rules. The study’s strength is that it uses data mining techniques to discover the core reasons. However, with a large dataset, both approaches, DT and artificial neural network, cannot perform well [14]. Traditional algorithms do not scale well with huge data and high-dimensional datasets. Similarly, data collected for investigation are not enough to conclude because it is limited to a single ward and one hospital and results could not be generalized. In real-life scenarios, preprocessing of data is obligatory for the implementation of data mining approaches and to avoid biases in results.

Satti et al. [15] employed machine learning methods such as logistic regression, RF, DTs, and support vector machines to analyze infant mortality in Rwanda. It is suggested that machine learning be used to target various health outcomes, such as extremely preterm survival, neonatal death, stunting, and low birth weight newborns. Studies [16,17] used a classification stacking model to categorize the four main neonatal diseases: sepsis, birth asphyxia, necrotizing enter colitis, and respiratory distress syndrome. Most neonatal deaths are caused by these diseases. The dataset was gathered between 2018 and 2021 from the Asella Comprehensive Hospital. The created stacking model was contrasted against the XGBoost (XGB), RF, and support vector machine learning models. The study aids in the early identification and precise diagnosis of neonatal diseases, particularly for healthcare facilities with limited resources.

It can be observed from the literature that multiple statistical approaches were used to identify risk factors of child mortality for intervention and to predict mortality with assumption-based algorithms. In resource-limited countries, providing decision support systems to health professionals and health facilities with insufficient resources to investigate the likelihood of child mortality is essential to minimize child mortality and achieve SDGs provided by the United Nations. Healthcare data are increasing at an implausible proportion, and developing prediction algorithms with scalability in mind is a vital strategy constraint. Therefore, the study contributes some value to the improvement of childhood health by analyzing the child mortality risk factors with the help of the feature selection method, the information gain, and the predictive analytics framework designed for the prediction of child mortality.

## 3 Materials and methods

Section 3.1 describes the study area and how data are collected for the development of the predictive model. Section 3.2 presents the framework for the prediction of child mortality. Section 3.3 demonstrates how data are processed and which risk factors are critical for the estimation of child mortality. Section 3.4 describes the supervised learning algorithms that are considered for model development. Section 3.5 concludes optimal model selection based on performance evaluation metrics.

### 3.1 Study area and design

In this study, we have used PDHS 2017–18 publicly available dataset. The demographic and health surveys are liable for gathering information on the well-being of the population from developing countries and this information can be freely downloaded from the MEASURE DHS database [6]. In Pakistan, two stages of stratified random sampling were used for the selection of households. About 580 enumeration areas (EAs) were chosen in the first stage and 561 EAs were successfully surveyed. In the next stage, the rest of the households are selected to deliver trustworthy estimates for the country. The attributes pertinent to the mortality of children under the age of five were extracted from the huge volume of the PDHS dataset. The dataset consists of 12,479 children from all over Pakistan. The critical socioeconomic and demographic risk factors influencing child mortality in Pakistan are included in Table 1.

**Table 1:** Risk factors of child mortality in Pakistan

Risk factors	Type	Distinct values
Age of mother at first birth	Numeric	30
Mother’s age	Numeric	35
Breastfeeding	Categorical	2
Father’s education	Categorical	2
Gender of the child	Categorical	2
Preceding birth interval	Numeric	146
Occupation of mother	Categorical	2
Education of mother	Categorical	2
Received family planning	Categorical	2
Residence	Categorical	2
Presence of diarrhea	Categorical	2
Child size at birth	Categorical	3
Region	Categorical	8
Wealth index	Categorical	3
Birth order number	Numeric	15
Antenatal care visits	Numeric	20
Place of delivery	Categorical	3
Child is twin	Categorical	2
Baby postnatal check-up	Categorical	2
No. of under-five children in a household	Numeric	13
Total children ever born	Numeric	15
Iron folic acid supply during pregnancy	Categorical	2
Family members	Numeric	38
Births in the last 5 years	Numeric	5

### 3.2 Proposed framework for predicting child mortality

**Figure 1:**
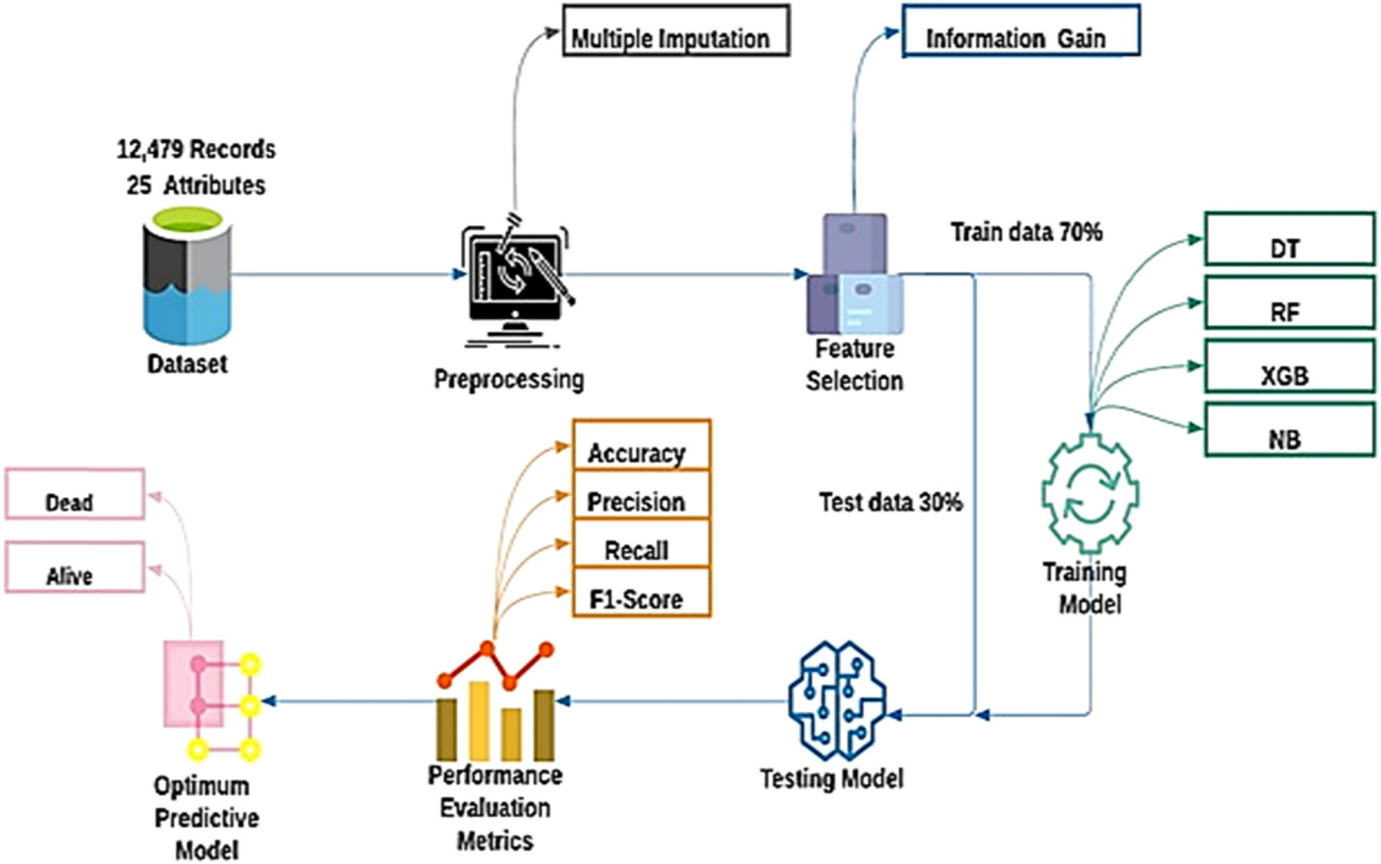
The proposed framework for predicting child mortality.

Following data retrieval, missing values are handled and we apply the information gain method on pre-processed data to rank the features with high information. Figure 1 illustrates the proposed framework. After implementation of the information gain method, the dataset is split into train and test sets. The objective behind splitting the dataset is that the machine learning classifier learns patterns from the training dataset and classifier performance is evaluated on the test dataset. We use various supervised machine learning classifiers for training and to evaluate the performance of classifier metrics like accuracy, precision, recall, and *F*1 score. Eventually, the machine learning algorithm that provides the most efficient results is selected. Ensemble learning is the best approach because it works well with different types of data in production. The efficiency of classifiers is compared and the one with the best results is adopted for the final prediction.

### 3.3 Preprocessing of data

Following data retrieval, data must be pre-processed by applying various data cleansing procedures. We use the predictive mean matching (PMM) algorithm in the SPSS tool to manage missing values during the pre-processing of data. After handling missing values, the next step is to identify critical risk factors from the dataset using the information gain selection technique. Information gain reveals the relative importance of a specific feature vector attribute. Later, we randomly split 70% of the dataset for training and the other 30% for testing, respectively. In pre-processing, another important task is to balance the distribution of the target variable. We applied the synthetic minority oversampling technique (SMOTE) to balance the dataset and eliminate sampling flaws because the sample sizes of the “Alive” and “Dead” subgroups were not distributed equally [9]. SMOTE produces artificial samples for the minority class by synthesizing the instances.

### 3.4 Model development

The practice of classifying objects into groups or categories based on a shared characteristic is known as classification. By learning from the training data, the classification approach builds a model. The model is utilized to categorize new objects. In this study, we have used DT, RF, NB, and XGB for the prediction of model development. In a classification problem, the strategy related to DT is the most constructive. The DT uses a tree-like structure and is most critical in classification problems. Using this approach, a tree is created to model the classification process. After the formation of the tree, it is applied to each record in the dataset and as a result, it suitably classifies the tuple. DT generates decision rules that help in discovering hidden patterns in a dataset.

To see which classifier works better on the selected dataset, RF is also used to categorize the labels of the classes. RF is a kind of ensemble technique in which different learning models are merged to enhance the generalization process. The perception behind ensemble lies in the fact that a pool of simple models could provide considerably better performance in comparison with a unique complex model that could be prone to over-fitting for its high variance. RF realizes an ensemble of DTs, while a tree characterizes a decisional method such that at each node a decision related to branching is taken by assessing the value of one feature against the threshold. Both the structure and the threshold of the tree are maintained during the learning process [18,19]. The RF builds several DTs, which are trained on randomly chosen subsets of training samples and the features of data, assembling their predictions to give the resultant ensemble output.

NB is one of the easiest and simplest algorithms for predicting results. It is based on the Bayes theorem, which states that one feature’s presence is completely independent of another feature’s presence. This uses a similar approach to estimate the probability of different classes based on different attributes. We applied NB because this algorithm makes it easy and efficient to foresee the class of the test dataset. It proves well in multi-class predictions [20,21]. It also responds better than other models when assuming autonomy holds, such as logistical regression, and less training data are required. Furthermore, it can also work easily with missing values [22,23,24]. NB measures the posterior likelihood using the prior likelihood and adds probability, which is new evidence.

XGB is an ensemble method and implements the gradient boosting concept but is more regularized. XGB is designed to be highly efficient, flexible, and robust enough to support the tuning of parameters and can perform classification and regression [25]. It is a boosting technique, in which weak learners are turned into good learners. There are other benefits such as parallel processing, handling missing values, tree pruning, and regularization to evade over-fitting.

### 3.5 Performance evaluation metrics

After the models are trained, the next step is to analyze the performance of models on an unseen dataset. To select an optimal model for the prediction of child mortality, each model is evaluated against 30% of the data, called test data. We used various metrics for the evaluation of classifiers such as precision, accuracy, recall, and *F*1 score [26]. Accuracy, being the most significant performance benchmark, acts as a ratio of accurately predicted observations against the total captured observations.

Precision can be termed as the ratio of accurately predicted positive values divided by the total number of positive values predicted. It is also called the positive predicted value and is a measure of the exactness of classifiers. The number of true positives divided by the aggregate number of true positives and false negatives defines recall. It is also named sensitivity or true positive rate.

## 4 Results and discussion

**Figure 2:**
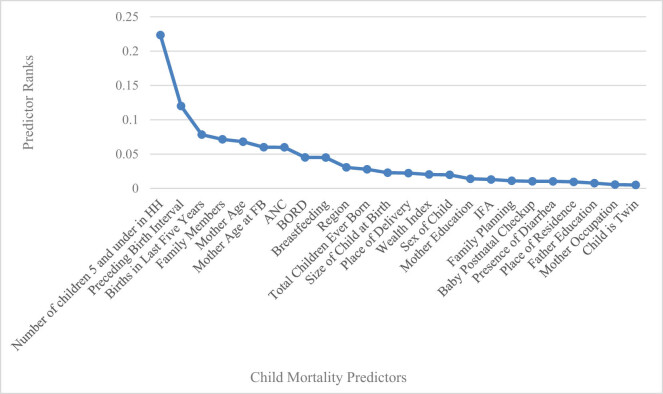
Features ranked according to information gain.

We discuss the experimental results provided by our established framework in this section. We have included 12,479 children under the age of five and selected 24 independent variables for model development. For smart data analysis, in this research, the feature selection approach, the information gain, ranked the predictors associated with under-five child mortality. Figure 2 shows information gain scores for predictors associated with child mortality.

It can be clearly observed that the feature selection method, the information gain, highlighted the same critical risk factors correlated with child mortality that we studied in the literature. Features highly correlated with label class are the number of under-five children in a household, preceding birth interval, births in the last 5 years, family members, mother’s age, age of the mother at first birth, antenatal care visits, birth order number, breastfeeding, region, total children ever born, size of child at birth, place of delivery, wealth index, and sex of child. Features ranked with less importance are the mother’s education and multiple births.

Patel et al. [3] show that healthcare professionals and the government of Pakistan are aware that birth spacing reduces child mortality. Similarly, mothers who use antenatal care facilities had a lower risk of their babies dying. The majority of investigations have discovered a strong association between breastfeeding and child mortality. According to Naz et al. [27], the children who were not breastfed had a greater risk of mortality compared to children who were breastfed. Research [9,10] conducted in developing countries also verifies our findings. According to previous studies [11,28], the under-five child mortality rate is not high in regions where prenatal and postnatal care facilities are available. Ahmed et al. [12] show that low birth weight is the leading cause of high mortality rates in Pakistan. According to our study findings, children born with a low birth weight have a higher risk of dying than the infant with normal weight at birth. Infants born at home have a higher risk of mortality before reaching the age of five than babies born in a public or private hospital, according to our findings. Naz et al. [29] depict that it is critical to have a professional health practitioner available during delivery to avoid child mortality. Another study [30] conducted in India shows that lack of adequate resources is characterized as poverty and causes high child mortality.

Later, we used 70% of the training data to create a predictive classification model. Table 2 shows that ensemble-based classifiers performed better than all other classifiers. RF is found to be the optimal algorithm with the highest accuracy (93.8%), precision (0.964), recall (0.971), and *F*1-score (0.967). XGBoost or XGB also performed well with accuracy (89.2%), precision (0.973), recall (0.911), and *F*1-score (0.941).

**Table 2:** Evaluation of classification models

Algorithms	Accuracy%	Precision	Recall	*F*1 score
DT	88.8	0.963	0.917	0.940
RF	93.8	0.964	0.971	0.967
NB	74.5	0.953	0.769	0.851
XGBoost (XGB)	89.2	0.973	0.911	0.941

Practitioners and researchers can use this paradigm to detect and predict mortality among children under the age of five using their datasets. Hence, our predictive analytic framework can assist health professionals to educate mothers as well as take preventive measures to reduce child mortality in resource-limited settings.

## 5 Conclusions

In this article, we exploited information gain to identify significant features for child death and established a predictive analytic framework using machine learning algorithms for the prediction of child mortality. Machine learning algorithms like DT, RF, NB, and XGB were tested on Pakistan demographic and health survey dataset, and discovered that RF with 93.8% accuracy surpassed the other classifiers.

The features include the number of under-five children in a household, preceding birth interval, births in the last 5 years, family members, mother’s age, age of mother at first birth, antenatal care visits, birth order number, breastfeeding, region, total children ever born, size of the child at birth, place of delivery, wealth index, and sex of the child are key risk factors and are directly connected with under-five child mortality, according to a smart analysis with information gain. In Pakistan, predictive analytics could enhance child health programs and notably help in advancing smart healthcare systems to estimate mortality patterns for timely intervention.

Mothers' recall bias in the reporting of events is the main limitation of this research. It is not possible for a mother to recognize events from the past. Similarly, due to the nature of the survey cause specific mortality among children under the age of five cannot be identified. AutoML can be used in the future to improve accuracy for the estimation of child mortality and to also reduce user-computer interaction while training the model.
